# Decoupled Visually-Guided Reaching in Optic Ataxia: Differences in Motor Control between Canonical and Non-Canonical Orientations in Space

**DOI:** 10.1371/journal.pone.0086138

**Published:** 2013-12-31

**Authors:** Joshua A. Granek, Laure Pisella, John Stemberger, Alain Vighetto, Yves Rossetti, Lauren E. Sergio

**Affiliations:** 1 School of Kinesiology and Health Science, Centre for Vision Research, York University, Toronto, Ontario, Canada; 2 Impact - Centre de Recherche en Neurosciences de Lyon, Inserm U 1028, CNRS UMR 5092, Bron, France; 3 Biologie Humaine, Université Lyon, Lyon, France; 4 Mouvement et Handicap, Hospices Civils de Lyon, Hôpital Neurologique Pierre Wertheimer, Bron, France; University of California, Merced, United States of America

## Abstract

Guiding a limb often involves situations in which the spatial location of the target for gaze and limb movement are not congruent (i.e. have been decoupled). Such decoupled situations involve both the implementation of a cognitive rule (i.e. strategic control) and the online monitoring of the limb position relative to gaze and target (i.e. sensorimotor recalibration). To further understand the neural mechanisms underlying these different types of visuomotor control, we tested patient IG who has bilateral caudal superior parietal lobule (SPL) damage resulting in optic ataxia (OA), and compared her performance with six age-matched controls on a series of center-out reaching tasks. The tasks comprised 1) directing a cursor that had been rotated (180° or 90°) within the same spatial plane as the visual display, or 2) moving the hand along a different spatial plane than the visual display (horizontal or para-sagittal). Importantly, all conditions were performed towards visual targets located along either the horizontal axis (left and right; which can be guided from strategic control) or the diagonal axes (top-left and top-right; which require on-line trajectory elaboration and updating by sensorimotor recalibration). The bilateral OA patient performed much better in decoupled visuomotor control towards the horizontal targets, a canonical situation in which well-categorized allocentric cues could be utilized (i.e. guiding cursor direction perpendicular to computer monitor border). Relative to neurologically intact adults, IG's performance suffered towards diagonal targets, a non-canonical situation in which only less-categorized allocentric cues were available (i.e. guiding cursor direction at an off-axis angle to computer monitor border), and she was therefore required to rely on sensorimotor recalibration of her decoupled limb. We propose that an intact caudal SPL is crucial for any decoupled visuomotor control, particularly when relying on the realignment between vision and proprioception without reliable allocentric cues towards non-canonical orientations in space.

## Introduction

The evolution of the human cerebrum has enabled us to interact indirectly with objects via the use of tools. Tool-use requires combining the semantic properties of the functionality of the tool, with the appropriate orientation of the distal musculature [1]. In addition, top-down control is needed to inhibit the natural tendency to directly interact with a viewed object [2-6]. The integration of an explicit cognitive rule with a motor action has been referred to as *strategic control* [7-9]. However, motor skills which require something other than direct object interaction (i.e. “standard” sensorimotor mapping) [10] also require a coordinated remapping between different sensory modalities such as vision and proprioception [11]. The adaptation by the brain to spatial orientation differences has been referred to as *sensorimotor recalibration* [12-14], and comprises a control mode that is more gradual and does not involve conscious awareness [15] (often referred to as ‘implicit’ learning). In both these explicit and implicit situations, a spatial algorithm must be integrated into the motor plan in order to accurately compute the relative positions of the visual cues with the required direction of the decoupled limb (i.e. “non-standard mapping”) [10], and are crucial for everyday activities such as using a computer mouse or driving a car.

There are a variety of motor behavioral tasks one may use to employ rule-based movement control in order to examine cognitive-motor integration. In the present study, each task was designed to involve a different *weighting* between strategic control and sensorimotor recalibration. The task manipulations, in which participants were instructed to foveate the visual targets, involved moving the decoupled limb to targets when there was either 1) a rotated cursor feedback between viewed hand motion and actual hand motion (180° and 90°), or 2) a change in the plane of the displayed visual stimulus (vertical) relative to plane of the limb movement (horizontal and para-sagittal planes). All conditions were performed both “head-fixed” (in a chin rest) and “head-free”. The head-free conditions were implemented in order to confirm that performance would be maintained during more “naturalistic” [16] and synergistic [17] head-eye movements. Further, the visual targets for the center-out reach task were placed either along either a *horizontal* axis (right or left) or along a *diagonal* axis (top-left or top right). Differences in learning behavior have been previously observed depending on target location. Participants learning a left-right inversion (similar to a 90° rotation) towards *diagonal* targets have been shown to initially reach to a direction that is opposite to the cued target, then gradually adjust the planned movement direction in order to compensate for the x-axis inversion [18]. In contrast, only a transient increase in variability was observed during a left-right inversion towards targets along ordinal (horizontal and vertical) axes [18]. 

Although an extensive cortical network for non-standard decoupled visuomotor control has been established using brain imaging [19-23], their distinct components (i.e. strategic control versus sensorimotor recalibration) have not been fully characterized. Our first objective in the current study was to determine if different cortical networks were involved in strategic control versus sensorimotor recalibration. To address this objective, we examined the performance on a series of decoupled eye-hand coordination tasks by an adult with bilateral caudal SPL lesions (patient IG) resulting in optic ataxia (OA), relative to neurologically intact adults. Patient IG has bilateral OA, predominantly known for her misreaching toward visual targets in peripheral vision, as well as a deficit of on-line hand motor control. Indeed, previous observations in OA have revealed that a damaged caudal SPL [24] can lead to deficits in online updating of limb position in eye-hand coordination [25-30] with visual field and hand effects [25]. OA patients typically misreach when guiding a limb in peripheral space towards extra-foveal targets [27,31] which has been shown to activate the parieto-occipital junction [32]. These deficits observed in OA extend to situations which require the peripheral guidance of a visible decoupled limb towards foveally-acquired visual targets [11]. 

Decoupled eye-hand coordination involves the ability to predict hand location without direct vision, and since impaired proprioceptive updating has been observed in OA (as seen in reaches in the dark) [33], patients may be required to rely on strategic control in such situations. In contrast, “standard” eye-hand coupling, which in the dark relies on the medial intraparietal sulcus (IPS) [32] also lesioned in most OA patients, is generally spared in conditions performed with illuminated vision of the hand [34-36]. However in a recent study [37], parietal patients recovering from brain tumor removal surgery have been shown to exhibit impaired foveal reaching accuracy compared with other brain tumor patients (premotor and prefrontal), and to display correlated reaching impairments between foveal and peripheral targets. On an individual basis, five out of the seven parietal patients demonstrating foveal reaching impairments also displayed contralesional hand effects [37], implying more anterior parietal lesions [24]. Similarly, right parietal patients following tumor removal surgery have been shown to display impaired mental rotations of a visually-displayed shape, specifically when relying on categorical rules (i.e. choosing an appropriate corner to use as guidance). Such an impairment would putatively contribute to previously observed deficits seen in OA during rotated visuomotor control [11].

Guiding a decoupled limb towards targets placed on a horizontal line relies on rule-based strategic control more than targets placed on a diagonal line. In our decoupled conditions, similar to the x-axis inversion performed in [18], one is able to rely on allocentric cues for movement guidance to horizontal targets, since a straight path to these targets is aligned with the horizontal borders of the target display monitor (and perpendicular to the vertical border just beyond the target). Use of allocentric cues have been shown to be important for visuomotor adaptations, which have been shown to be represented in extrinsic coordinates [38]. In addition, we have previously reported the reliance of additional saccades towards the computer monitor border (i.e. “hypermetric steps”) in unilateral OA towards horizontal and vertical targets [11]. In contrast, one would not be able to rely on allocentric cues to plan a straight path to targets placed diagonally from the central target since the computer monitor corner was not along a diagonal line from the start location. Hence although participants might have an approximate rule for the diagonal targets using allocentric cues (e.g. top-left target is close to a bottom-right movement), this rule could only be used as a guideline since the surrounding allocentric cues were not precise enough to devise a predictive motor plan; thus, a gradual recalibration between senses would be required. Comprehension of the cognitive rule has not been shown to be sufficient for successful adaptation in off-axis situations [39-41].

We have previously reported impaired performance in OA on visuomotor rotations relative to a spatial plane dissociation [11]. To extend this research, in the current study we have included a 90° visuomotor rotation condition. Movements in the opposite direction to the cursor feedback (involving the inversion of *both* x and y axes) in the 180° rotation condition are easier to learn via quick strategic control, while movements made under a 90° cursor feedback rotation (involving the inversion of *either* x or y axes) are more difficult to perform and rely heavily on gradual sensorimotor recalibration. Behavioral support for this assumption comes from previous studies in which neurologically healthy adults showed minimal behavioral performance degradation with a 180° cursor feedback rotation versus no rotation [42,43]. In contrast, it has been demonstrated that reach performance declines as feedback rotation moves from 0° to 90°, improves from 90° to 180°, and then declines again from 180° to 270° [39,44]. These data support the idea of two ‘functional modules’ or control modes [45], in which a simple ‘move in opposite direction’ requirement employs a quick to implement rule-based strategy while intervening angles – maximizing at 90° – employ a gradual recalibration [6,45]. Similarly, decoupled hand movements along the para-sagittal plane are often less familiar than decoupled hand movements along the horizontal plane (as in the use of a computer mouse. In our previous work with patient IG, she showed success during a horizontal spatial plane transformation [34], which may reflect cortical activation changes [22] as a result of previous experience with a similar decoupled task (e.g. using a computer mouse). In addition, improvements in grasping deficits have been previously observed in OA towards familiar objects [46]. Thus we also introduced a decoupling involving motion in a para-sagittal plane to reduce the effect of familiarity on movement performance. In summary, here we employ movements having varying levels of well-categorized canonical and less-categorized non-canonical orientations and directions, which thus follow a spectrum of strategic control to sensorimotor recalibration. 

As such, we hypothesized that an intact caudal SPL is crucial for situations relying on sensorimotor recalibration, but not for situations in which strategic control is more useful. From this hypothesis, we predicted that the damaged bilateral caudal SPL seen in patient IG would cause performance impairments in situations that relied predominantly on an implicit realignment of the decoupled proprioceptive and visual input. These performance impairments would be beyond the previously observed visuomotor deficits seen in OA such as slow and inaccurate motor predictions towards targets along the ordinal axes [11] and deficits in automatic online updating [29,30,47]. In contrast, we predicted that IG’s performance would improve, although still be compromised relative to controls [11], during decoupled movements guided by accurate allocentric cues in situations in which strategic control could be employed. 

Our second exploratory objective was to examine what the effects of decoupled eye-hand coordination were on motor error patterns in bilateral OA during situations where strategic control would be used versus situations where sensorimotor recalibration would be used. We hypothesized, based on our results with unilateral OA patients [11] that an intact caudal SPL formulates and maintains a "difference vector" between the felt hand and the viewed action goal only when relying on sensorimotor recalibration. Specifically, we predicted that IG would perform additional saccades and additional head movements (when allowed) in order to continually monitor and recalibrate the required difference vector between limb, gaze, and visual target locations during situations relying primarily on sensorimotor recalibration towards non-canonical orientations in space. 

## Methods

All participants signed informed consent and the study protocol was approved by the York University human participant research ethics committee.

### Participants

The participants tested in the current study were one patient with dorsal visual stream damage (IG, age 44) and six healthy age-matched controls (three females), mean age 39 ± 9. All participants were tested using their dominant, right hand (handedness score greater than +0.50) [48] and had experience with a computer mouse and laptop touchpad.

### Patient details

**Figure 1:**
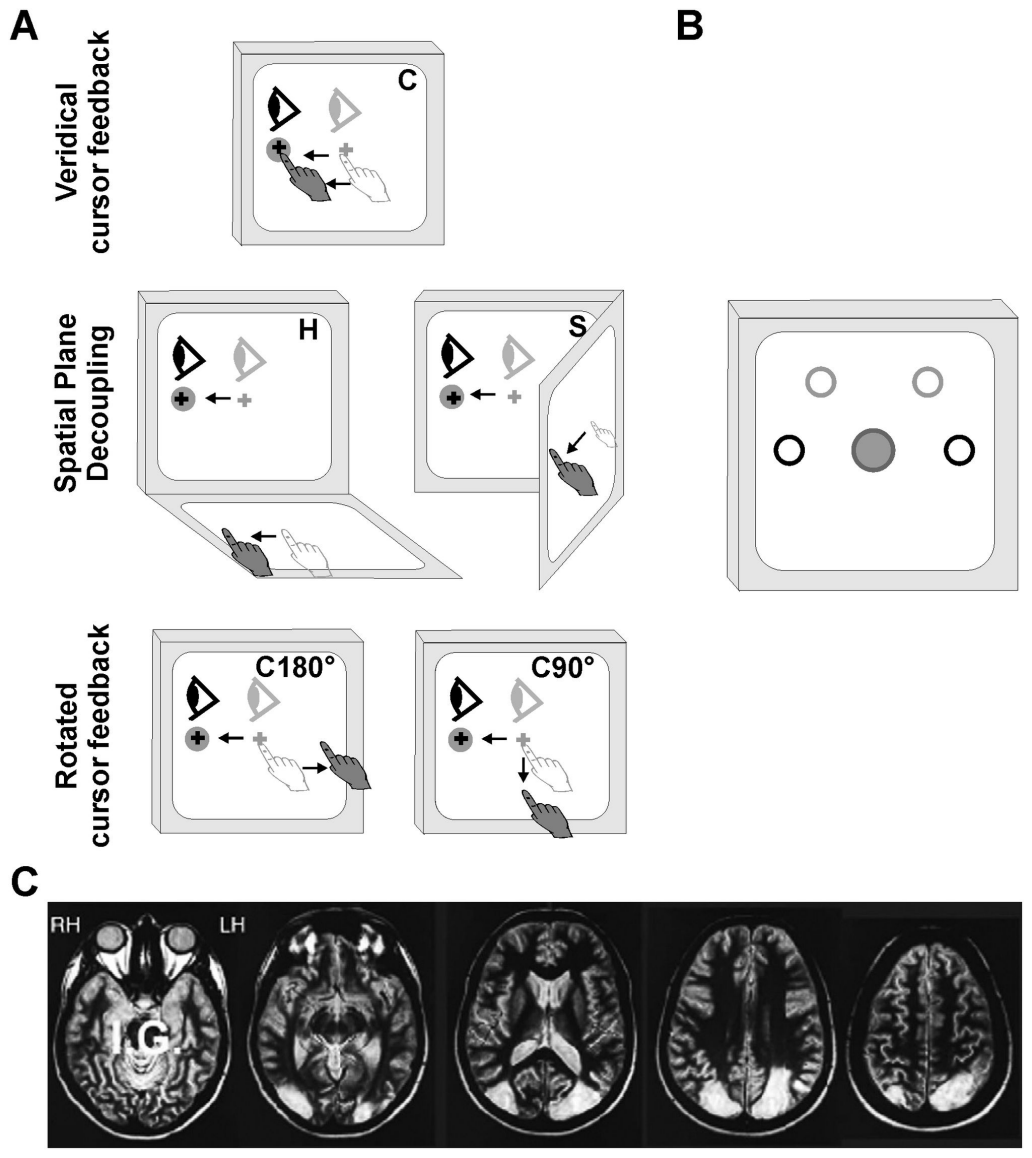
Task procedure, anatomical MRI scan slices. (A) Schematic drawing of the standard center-out reaching movement (C, coronal) and the two basic manipulations: spatial plane (H, horizontal; S, para-sagittal) and cursor feedback rotation (C180°/90°). The visual stimuli were always presented on the vertical monitor. The gray circles denote the cued position before the movement. Light eye and hand symbols denote starting positions. Practice trials were performed before each condition (presented in randomized order) until it was reported that the task was sufficiently familiar for testing to begin. All conditions were performed both head-fixed and head-free in addition to a control eye only (head-fixed) and a control gaze only (head-free) condition. (B) Schematic of horizontal (black) and diagonal (gray) target locations. Note that targets in lower hemifield were not tested to avoid IG's scotoma. (C) Axial T2-weighted MRI scans of IG's cortex revealed fairly symmetrical lesions located in the posterior parietal and upper and lateral occipital cortico-subcortical regions (mainly BA 19, 18 and 7, and a limited part of area 39 and the IPS).

At the time of testing, IG was a 44 year old woman who suffered from an ischemic stroke related to acute vasospastic angiopathy in the posterior cerebral arteries established with an angiogram. Magnetic resonance imaging revealed a hyperintense signal on T2 sequencing that was fairly symmetrically, located in the posterior parietal and upper and lateral occipital cortico-subcortical regions (Figure 1C). Reconstruction of the lesion [49] indicated that it involved mainly Brodmann’s areas 19, 18, 7, a limited part of area 39. This therefore included the parieto-occipital sulcus and the caudal part of the intraparietal sulcus. IG was given a set of standard clinical tests involving visual field topography (Goldman perimetry), sensory stimulation tests (visual and tactile extinction), neurological evaluation of reflexes and muscle tone and joint movements. Visual fields showed a partial right inferior homonymous quadrantanopia (visual scotoma). The patient did not exhibit any purely motor or somatosensory deficit, any sign of sensory extinction or any sign of neglect during conventional testing (on standard line bisection, star cancellation and drawing tasks) but she demonstrated bilateral optic ataxia [24,50]. OA patients typically display in impaired online peripheral guidance of a limb [27,31] with improved accuracy when given more processing time as in delayed reaching [51], along with preserved low-level visual and motor function [34-36]. Reaching and grasping inaccuracy predominated for her right hand in her right peripheral hemifield. However, visually elicited hand movements were generally accurate when performed in foveal vision. Note that IG initially showed simultanagnosia, which prevented her to see the target and her hand at the same time, which could lead to a contradictory result [52]. In the present experiment, however, IG was tested when the bilateral optic ataxia persisted without associated clinical simultanagnosia.

### Experimental procedure

Participants sat in a darkened room in front of a computer monitor placed 41 cm away, aligned with their mid-sagittal plane. They made sliding finger movements over a touch sensitive screen (Keytec Magic Screen: Model KTMT-1315: Sampling rate: 100 Hz) in order to displace a cursor from a central target to one of two horizontal (right or left), or to one of two diagonal (45° rotated from a vertical line – approximately top-right or approximately top-left) targets. Importantly, contrary to the horizontal targets which are oriented directly perpendicular to the dimly lit computer monitor border, the diagonal targets were not oriented directly towards any helpful allocentric cues such as the corner of the computer monitor. Following a two second delay, one of the peripheral visual targets (16mm in diameter) was presented 110 mm (15° visual angle) from the central target (25mm in diameter), always on the vertical monitor. Participants were trained to move the cursor as accurately and quickly as possible across the touch screen into the target, and were encouraged to maintain a consistent initial arm orientation for the different task conditions of the experiment. In addition, all participants were instructed to perform a saccade towards the peripherally-cued visual target and to maintain fixation of the target until the end of the trial. Eye movements were monitored at 250 Hz (right eye, Cambridge Systems and EyeLink II). The viewing space was calibrated using a nine-point calibration and drift correction was applied between each condition. 

Figure 1A displays a schematic of all of the experimental conditions. The participants performed a single standard condition and four decoupled conditions. There were two ways in which the visual target could be decoupled from the required hand movement: a change in the spatial plane of the hand movement or a change in the motion of the viewed cursor relative to the motion of the hand (rotated visual feedback). In the standard condition and the rotated visual feedback conditions, the touch screen was placed over the computer monitor (C, in a coronal plane). The touch screen was also placed in two other spatial planes: horizontal (H, lying flat in front of the participant aligned with their midline) and para-sagittal (S, affixed to a custom mount in line with the subject’s right shoulder). The para-sagittal plane was chosen to ensure that control of a spatial plane dissociation was not aided by previously learned rules involving a computer mouse and/or laptop touchpad (such as in H). Thus, the horizontal and para-sagittal planes represented two decoupled conditions, since the visual targets were always presented on the computer monitor. Within the coronal plane (where the touch screen was placed on the monitor), cursor feedback rotation was altered either 180° (e.g. move hand leftward to displace cursor rightward) or 90° clockwise (e.g. move hand upward to displace cursor rightward). Thus the cursor feedback rotations in the plane of the computer monitor represented the other two decoupled conditions, C90° and C180°. The C90° condition was used to present a situation where a simple rule could be implemented for the horizontal targets (e.g. right = down), but a more implicit mental rotation of the cursor was required for the diagonal targets. In order to test for effects of head restraint, each condition was performed with the head being restrained using a chin rest (head-fixed), and with the head not restrained (head-free). 

In order to ensure equal understanding of the transformational rules applied in each condition, all participants were trained prior to each randomly assigned block until each subject reported that they were adequately prepared. IG felt cognitively prepared following training for a total of six trials for C, 12 for H, 27 for S, 30 for C180°, and 40 for C90°. The control subjects reported to adequately prepared following training for a mean total of 12.2 trials for C, 22.6 for H, 30.6 for S, 21.2 for C180°, and 37.7 for C90°. Following training, each participant performed 40 trials (20 head-fixed trials, 20 head-free trials) in each of the five experimental conditions (Figure 1A). In addition, in order to ensure proper oculomotor control in the patient, participants performed a sixth condition in which all participants performed 40 saccades (20 eyes-only trials, 20 gaze-only trials) towards the peripherally-cued targets without any hand movements. 

For the head-free conditions, the relative changes in absolute translation and roll of the head were extrapolated from the change in position of an infrared camera positioned on the middle of the forehead (Cambridge Systems and EyeLink II) relative to four infrared calibration points. These relative changes in translation and roll were verified with video from a head-mounted video camera. In order to emulate a natural environment, all subjects were instructed to look at the visual target (i.e. foveal acquisition), but were not restricted to a certain eye path. In the darkened room, the border of the computer monitor and the hand were still visible with peripheral vision. 

### Data Analyses

We calculated an index reflecting the overall performance decrements observed during eye-hand decoupling for patient IG relative to the control group. The index was computed as the mean number of standard deviation units IG differed from the control group (i.e. *effect size*; ES) for the 17 hand and eye timing, trajectory and error variables tested (not including summary variables; see below for details). ES was calculated for each trial as a change in each decoupled condition (H, S, C180°, and C90°) relative to the standard condition for each participant, and for each target type (horizontal versus diagonal). Importantly, a positive effect size represents an increased impairment for IG relative to the controls. For example, a *slower* peak velocity of the hand movement for IG relative to the controls would be calculated as a *positive* ES.

Trials were only included in the hand and eye movement analyses if they were successfully completed within a maximum of ten seconds and performed without an initial hand direction error (DE). A DE was quantified as a hand movement that deviated greater than 45° to either side of a straight line between the central and peripheral target for three consecutive time bins occurring in the first half of the ballistic movement. Although trials including these initial miscalculations were not included in further analyses, the number of DE were calculated for each participant in a separate analysis. For each DE, we also calculated the time to recovery (TTR), which was recorded from the time from the inaccurate hand movement onset (see below) until the time point in which the trajectory was reversed towards the correct target location.

The individual hand movement data were first low-pass Butterworth reverse filtered at 10 Hz (Matlab, Mathworks Inc.). Hand movement timing was analyzed whereby hand reaction time (HRT) began when the peripheral target was presented and ended at movement onset. Hand movement onsets were scored as the point at which in which the tangential velocity exceeded 10% of its peak using a custom-written computer algorithm. The hand ballistic movement time (*HMT*) for all conditions began from the hand movement onset and ended at the first point in which the movement slowed to 10% peak velocity. The automatically scored onset and offset was verified visually for each trial (before any overlapping corrective movements). In order to quantify the timing for corrective movements, we analyzed corrective movement time (CMT), which began at the end of HMT (10% peak velocity) of a given trial and ended when the cursor entered the perimeter of the peripheral target (trial completion). For summary purposes, we also report total movement time (TMT), which began at hand movement onset and ended when the cursor entered the perimeter of the peripheral target. *Peak velocity* was recorded as the maximum tangential change in resultant x and y position over time between movement onset and when the cursor entered the perimeter of the peripheral target. Path linearity was measured using hand movement paths (*path length*), which were recorded as the distance travelled from movement onset to when the cursor entered the perimeter of the peripheral target. In addition, the absolute angle (in degrees) of the vector from the starting point to the point of the trajectory that corresponds to the maximum velocity relative to a straight line between the central and the peripheral target was recorded for each trial (*angle at peak velocity*). Hand movement accuracy parameters were determined from the participant’s mean movement endpoints for each target location and analyzed separately for distance errors (*on-axis CE*) and for direction errors (*off-axis CE*). Endpoint precision (variable error, *VE*) was determined by the distance of the endpoints of the individual movements from their mean movements. For summary purposes, we also report absolute error (AE), which was determined as the absolute value of each participant's mean movement endpoint errors for each target location. 

Eye position data were first low-pass Butterworth reverse filtered at 50 Hz (Matlab, Mathworks Inc.) and were drift corrected prior to each trial. Eye movement timing was analyzed whereby eye reaction time (ERT) began when the peripheral target was presented and ended at saccadic onset. Eye movement onsets were scored as the point at which the resultant of the x and y trajectories exceeded 10% of the peak velocity. Eye movement time began at saccade onset and ended when the pupil entered the perimeter of the peripheral target.

Eye scan paths were recorded in order to observe the un-restricted eye movement behavior when the hand was spatially decoupled from gaze direction. The eye scan paths were only analyzed for a given trial if the corresponding hand movement trial was successfully completed. Each sampled data point obtained during the experiment that was registered as a blink was interpolated off-line using data obtained from the nearest accurate measurement before and after the point. Blinks were detected from a transient reduction in the pupil size measurement, provided by the eye tracking system. In order to be able to identify saccade-related errors, eye scan path data were recorded from eye movement onset until hand movement onset (early errors: “priming”) and from hand movement onset until entrance of peripheral target (late errors: “online updating”). The saccade-related errors were placed into three categories: 1) *steps* 2) *look-backs*, and 3) hand-biased mis-saccades (HBMS). Saccade-related errors were only coded if they occurred greater than 10% (11 mm) of a full saccade (from central to peripheral target) from the target border to ensure we were not enumerating eye movements within the target. The resulting errors were categorized as steps if an eye movement trajectory continued for at least 100 ms. *Hypometric* steps were defined as brief saccadic pauses occurring before reaching the peripheral target, while *hypermetric* steps were recorded when these small saccadic pauses occurred beyond the peripheral target towards the border of the computer monitor. Look-backs were counted when subjects reversed eye direction (towards the cursor) a minimum of 20% (22 mm) of the total amplitude from the central to peripheral target, holding at least 100 ms. HBMS were recorded if the initial and/or final saccadic endpoint was biased (greater than 10% of total distance from central to peripheral target) towards the direction of the hand during the decoupled conditions. 

### Statistical analyses

In order to determine if successful learning occurred following training in each task (albeit not necessarily complete visuomotor adaptation), initial paired t-tests were performed for each participant between the first five trials and the last five trials performed for each condition and each target type. After confirming a performance plateau for all participants, the data from the individual patient and the control group were analyzed separately. To screen for the effects of head movement (head-fixed versus head-free) on each condition, we initially conducted three-way repeated measures ANOVAs with condition, target type (horizontal versus diagonal targets), and head movement as within-subject factors on the control group. For patient IG, we initially conducted fixed-effect intra-subject ANOVAs also with condition, target type and head movement as within-subject factors. No condition × head-movement interactions were observed following either statistical test. Therefore, all further analyses were pooled across head-movement conditions for each task condition. In addition, we also initially screened for an effect of timing (i.e. "priming" versus "online updating") depending on condition and target type. Overall, since IG’s baseline data (i.e. direct visuomotor control) did not differ from the control group for any dependent variable (*p*′>0.05; see explanation for modified t-test performed below), all further analyses focused on the eye-hand “decoupling” (i.e. decoupled – coupled) aspect of non-standard visuomotor control. Specifically, IG’s data was presented as the mean change in her “decoupled” relative to her “coupled” performance for each condition and each target type. For the control group, eye-hand decoupling was determined as the relative change in performance between decoupled and coupled reaching for each dependent variable and each target type. In order to control for baseline differences across control subjects, we statistically removed (i.e. covaried for) the effects of the coupled task from that of the decoupled tasks. All repeated measures ANOVA results were reported with Greenhouse-Geisser-corrected p-values, and all post hoc comparisons were corrected for multiple comparisons (Bonferroni).

Inter-group analyses between IG and the control group were performed using modified t-tests [11,53] for each condition and for each visual target type. Importantly, for accurate comparison of a case to a control group, the modified t-tests utilized in the current study adjusted the critical t-value depending on the variability (standard deviation) and group size of our control group (for details, see 53). Therefore, alpha levels for all inter-group analyses were adjusted to 5% at *p*′<0.05 [53]. Importantly, the corrected alpha level for each modified t-test was then corrected for multiple comparisons (Holm-Bonferroni). In addition, an index of the number of standard deviation units that IG's score differed from a randomly chosen control subject (“effect size”) was calculated for each modified t-test to demonstrate the magnitude of the difference between groups [53]. One exception was during the comparison of the change in the number of eye “look-backs” between IG and the control group. In this case, the control group did not perform such errors (mean 0 ± 0), and therefore, no statistical comparison could be performed. In order to assess the level of eye-hand coupling in both IG and our control subjects, separate correlation analyses were performed between the eye and the hand reaction times (ERT and HRT) for the both the direct and the decoupled conditions.

## Results

Following training, no differences (*p*>0.05) in timing, trajectory and endpoint variables were observed across participants between the first five trials and the last five trials performed for each condition and target type. Having established that motor performance had reached a plateau, we could then assess cognitive-motor integration accurately. Further, to determine the effect of head restraint in an experimental setting, we tested all conditions with both head restraint (head-fixed) and head movement (head-free). Importantly, for all of the dependent variables tested, no task condition × head condition interactions were observed within the control group or within intra-subject analyses for patient IG (*p*>0.05). Therefore, all inter-group analyses were pooled across both head conditions (head-fixed and head-free) for each task condition. In addition, IG’s control condition performance (eye movement and gaze without hand movement) and standard eye-hand coordination performance (i.e. direct interaction with the viewed target) did not differ from the control group across all tested dependent variables. Thus, IG’s oculomotor control was not compromised and she was able to look at and reach directly to a freely viewed target without difficulty, similar to our control participants. Therefore, since both the patient and control groups performed at a similar level in these standard situations, we focused our analyses on the visuomotor control of the decoupled eye-hand movements (see Methods for details).

### Effect size

**Figure 2:**
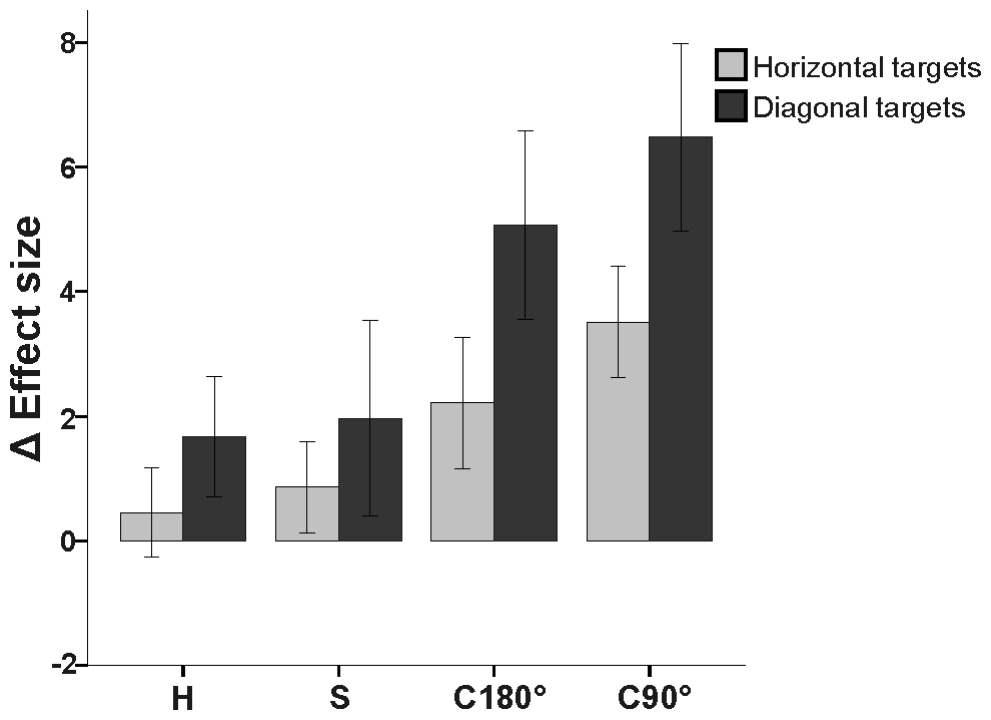
Effect size for decoupled conditions between IG and the control subjects. Mean number of standard deviation units (i.e. effect size; ES) in which IG differed from control group for each dependent variable during the decoupled conditions by target type (horizontal = light gray, diagonal = dark gray). Note the increase in ES for the diagonal targets relative to the horizontal targets, and for the rotated visuomotor tasks (C180° and C90°) relative to the spatial plane dissociations ( H and S). Error bars denote 95% Confidence Intervals.

We calculated an index reflecting the overall performance decrements of eye-hand decoupling for patient IG relative to the control group (i.e. change in effect size; ES, see Methods for details). We observed both condition (ANOVA, F_3,2255_ = 19.6, *p*<0.0001) and target (ANOVA, F_1,2255_= 23.0, *p*<0.0001) main effects for ES. Similar to previous results in unilateral OA [11], IG's performance was the most compromised relative to the control group for the visuomotor rotations (C180° and C90°). Importantly, we expand these previous findings by observing that IG’s performance was significantly more compromised towards the diagonal targets relative the horizontal targets (*p*<0.0001, see Figure 2). Specifically, post hoc analyses revealed a greater ES for C90° relative to H and S (*p*<0.0001), and C180° relative to H (*p*<0.0001) and S (*p*<0.001). For details on the dependent variables comprising the ES, see below.

### Hand and eye movement timing

**Figure 3:**
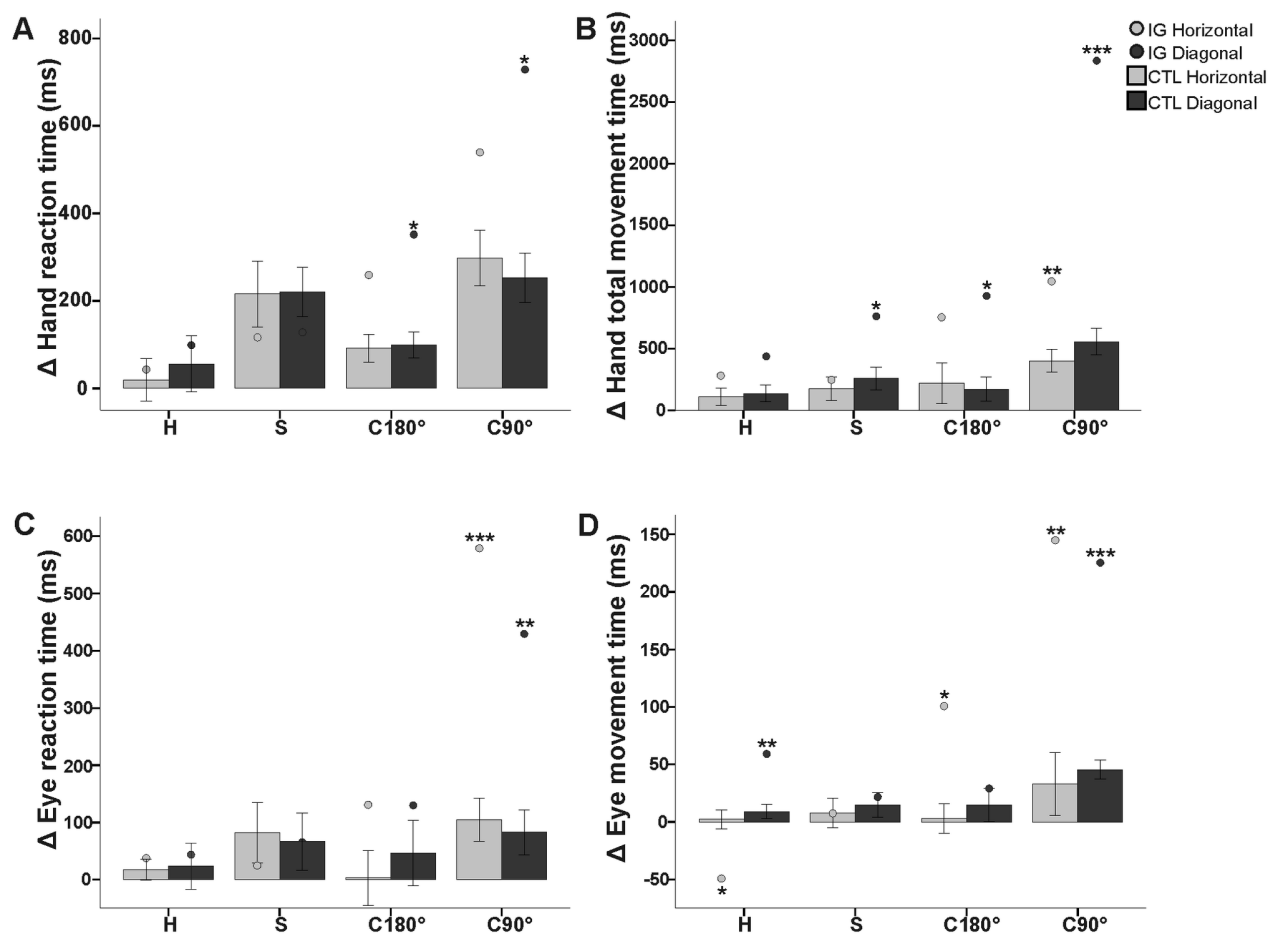
Hand and eye movement timing data for IG and the control group. Mean change in (A) hand and (C) eye reaction times and (B) hand and (D) eye total movement times in ms for both groups for the four decoupled conditions (H = horizontal; S = para-sagittal; C180° = coronal 180°; C90° = coronal 90°) relative to standard reaching for each target type (horizontal versus diagonal). Both head-fixed and head-free conditions were pooled for each subject. Error bars denote 95% Confidence Intervals. *p′<0.05; **p′<0.01; ***p′<0.001, corrected for multiple comparisons (Holm-Bonferroni).

In order to assess both predictive and online updating deficits as a result of OA, we analyzed eye and hand movement preparation and execution. Figure 3 shows the overall changes in hand and eye movement timing from baseline (direct, standard visuomotor control) for all subjects across the four decoupled conditions. For details on hand movement timing differences between IG and the control group depending on condition and target location, see Table 1. 

**Table 1:** Hand movement significant differences between IG and the control group separated by visual target type for each condition.

**Target type**	**Dependent Variable**	**Condition**	**IG: mean Δ in performance**	**Controls: mean Δ in performance (±95% CI)**	**t-value**	**Effect size**
Diagonal	HRT	C180°	354.4 ms	99.5 ± 31.9 ms	5.0*	5.4
		C90°	733.5 ms	253.0 ± 57.0 ms	5.6*	6.1
	HCMT	C180°	447.2 ms	46.6 ± 24.4 ms	10.8***	11.7
		C90°	2487.8 ms	309.1 ± 126.0 ms	15.4***	16.6
	Peak velocity	C90°	-69.0 ms/s	-4.5 ± 8.3 mm/s	-6.8**	-7.4
	On-axis CE	C180°	-12.1 mm	0.3 ± 1.0 mm	-10.2***	-11
	Off-axis CE	S	6.5 mm	0.8 ± 0.9 mm	4.7*	5.1
		C180°	6.2 mm	-1.0 ± 1.1 mm	6.3*	6.9
	Path length	C180°	32.6 mm	14.6 ± 6.6 mm	10.6***	11.4
	Angle	C180°	11.6°	2.4 ± 1.4°	5.5*	5.9
		S	12.2°	2.6 ± 1.1°	6.2**	6.7
		C90°	25.9°	3.1 ± 1.4°	17.1****	18.5
	TTR	C90°	2613.5 ms	410.5 ± 112.4 ms	18.1****	19.6
Horizontal	HMT	C180°	408.3 ms	52.6 ± 62.1 ms	5.1*	5.5
		C90°	316.6 ms	73.0 ± 77.0 ms	4.6*	5.0
	HCMT	C90°	702.7 ms	220.0 ± 85.7 ms	4.5*	4.8
		H	98.7 ms	26.2 ± 10.4 ms	4.8*	5.2
	Peak velocity	C90°	-57.0 ms/s	-21.5 ± 9.3 mm/s	-4.4*	-4.8
	Path length	C180°	24.0 mm	7.2 ± 3.7 mm	5.2*	5.6
	TTR	C180°	1622.0 ms	439.5 ± 0 mm	4.2*	4.5

Table 1 note: Dependent variables (HRT = hand reaction time; HMT = ballistic hand movement time; HCMT = corrective hand movement time; peak velocity; on/off-axis CE = constant error; Angle = angle at peak velocity; path length; TTR = time to recovery from a direction reversal) were tested with separate modified t-tests [53] for each condition (C = coronal 180°/90°; H = horizontal; S = para-sagittal) for each visual target type (diagonal/horizontal). Note that a negative change in peak velocity or on-axis CE reflects an impaired performance for IG relative to the controls. *p′<0.05; **p′<0.001; ***p′<0.001; ****p′<0.0001, corrected for multiple comparisons (Holm-Bonferroni).

#### Intra-subject OA details

Intra-subject analyses were conducted on patient IG as a measure of how difficult each decoupled condition was relative to her own standard performance, depending on target type. A condition main effect was observed for HRT (ANOVA, *F*_3,152_ = 30.3, *p*<0.0001) which revealed a greater impairment in hand-movement planning for C90° relative to all other decoupled conditions (*p*<0.0001), and C180° relative to H (*p*<0.01) and S (*p*<0.05). The ballistic motor performance (HMT) was the most compromised (condition main effect; ANOVA, *F*_3,152_ = 7.9, *p*<0.0001) for C180° relative to H (*p*<0.0001) and S (*p*<0.05). Meanwhile, online motor correction (HCMT) was the most impaired (condition × target interaction; ANOVA, *F*_3,152_ = 23.0, p<0.0001) for C90° relative to the other three decoupled conditions, specifically towards the diagonal targets (p<0.0001). IG's peak velocity was the slowest (main effect of condition; ANOVA, *F*_3,152_ = 2.8, p<0.05) during S relative to C180° (p<0.0001). In terms of eye movement timing, IG's eye movement preparation (ERT) was the slowest (condition main effect; ANOVA, *F*_3,131_ = 34.0, p<0.0001) during C90° relative the other decoupled conditions (p<0.0001). Similarly, IG's eye movement timing (EMT) was the slowest (condition main effect; ANOVA, *F*_3,131_ = 7.7, p<0.0001) during C90° relative to H (p<0.0001), S (p<0.001), and C180° (p<0.05).

#### Control group

Within-group analyses were conducted on hand and eye movement timing decrements within the control group in order to determine a baseline of difficulty depending on the condition and the target type. Condition main effects were observed for hand movement preparation (HRT; ANOVA, *F*_2,6_ = 18.9, *p*<0.01), and online movement correction (HCMT; ANOVA, *F*_2,8_ = 20.8, *p*<0.001). Similar to patient IG, post hoc comparisons revealed longer HRT for C90° compared with C180° and S compared with H (*p*<0.05), as well as longer HCMT for C90° relative to C180°, and H (*p*<0.05), and C90° relative to S (*p*<0.01). In contrast with patient IG, target orientation did not influence the hand movement timing parameters for each condition within this control group (*p*>0.05). Eye movement timing analyses within the control group revealed a condition × target type interaction for eye movement preparation (ERT; ANOVA, *F*_2,7_ = 5.8, *p*<0.05) and a main effect of condition for eye movement execution (EMT; ANOVA, *F*_2,8_ = 12.9, *p*<0.01). Post hoc comparisons revealed longer ERT for C90° compared with C180° and H for horizontal targets, as well as longer EMT for C90° relative to H across both target types (*p*<0.05).

#### OA patient versus control group

Relative to the control group, we observed different patterns of performance difficulty for the different types of decoupling (plane change, feedback rotation) presented to IG. Across target types, IG displayed longer HRT than the control group did for both conditions involving rotated cursor feedback (C180°/C90°: *t* > 4.2, *p*′<0.05, effect size > 4.5). For C90°, IG displayed the greatest increase in HRT than the control group towards the diagonal targets (see Table 1). IG also displayed an overall deficit (across target types) for total movement execution (TMT), relative to the control group, for C90° (*t* = 18.1, *p*′<0.0001, effect size = 18.1; see Figure 3B). The differences between groups in C90° were predominately comprised of increased hand movement timing during the corrective phase (HCMT) towards the diagonal targets (Table 1). These deficits in movement timing can also be explained in terms of hand movement velocity. Across targets, IG’s peak velocity was the most compromised, relative to the control group, during C90° (*t* = 7.4, *p*′<0.01, effect size = 8.0), whereby she slowed down the most towards the diagonal targets (Table 1).

Similarly, eye movement timing analyses revealed an overall decline in performance for patient IG relative to controls for C90°, although target type was not as large an influence on her deficits (Figures 3C,D). For the performance of C90°, IG displayed an overall greater decline than the control group did for eye movement preparation (ERT: *t* = 11.8, *p*′<0.001, effect size = 12.7) and for eye movement execution (EMT: *t* = 10.3, *p*′<0.001, effect size = 11.2). For details on eye movement timing between conditions and target types see Table 2.

**Table 2:** Eye movement significant differences between IG and the control group separated by visual target type for each condition.

**Target type**	**Dependent Variable**	**Condition**	**IG: mean Δ in performance**	**Controls: mean Δ in performance (±95% CI)**	**t-Value**	**Effect size**
Diagonal	ERT	C90°	429.9 ms	82.8 ± 39.1 ms	7.3**	7.9
	EMT	H	59.1 ms	9.0 ± 8.3 ms	7.2**	7.8
		C90°	225.2 ms	45.5 ± 8.2 ms	15.6***	16.8
	Look-backs	S	0.25/trial	0 ± 0/trial	+	+
		C180°	0.2/trial	0.01 ± 0.07/trial	6.1**	6.6
		C90°	1.5/trial	0.06 ± 0.03/trial	10.4***	11.2
	HBMS	C90°	0.28/trial	0.02 ± 0.03/trial	7.1**	7.7
Horizontal	ERT	C90°	577.5 ms	104.6 ± 37.7 ms	11.8***	12.8
	EMT	H	***-49.2ms***	***2.4 ± 8.3 ms***	-5.2*	***-7.4***
		C180°	100.7 ms	2.8 ± 12.9 ms	5.5*	5.9
		C90°	224.9 ms	32.9 ± 27.4 ms	6.2**	6.7
	Hypermetric	C180°	0.25/trial	-0.02 ± 0.03/trial	4.5**	4.9
	Look-backs	S	0.28/trial	0.0008 ± 0.03/trial	7.7**	8.3
	HBMS	S	0.17/trial	0.02 ± 0.03/trial	4.2*	4.6

Table 2 note: Significant differences (p′<0.05) between IG and the control group for each eye movement variable (ERT = eye reaction time; EMT = eye movement time; Hypermetric = hypermetric steps; HBMS = hand-biased mis-saccades) for each visual target type (diagonal versus horizontal). Modified t-tests [53] were performed on the relative changes for IG and the control group (± 95% CI) from decoupled to simple for each decoupled condition (H = horizontal; S = para-sagittal; C180° = coronal 180° rotated; C90° = coronal 90° rotated). Bold/italics imply control group performed worse than IG. + No statistical comparison between the case and the control group could be performed because the control group had a mean and standard deviation of zero. *p′<0.05; **p′<0.001; ***p′<0.001, corrected for multiple comparisons (Holm-Bonferroni).

In summary, decoupling the spatial location of the foveally-acquired visual target and the hand motion required to reach that target led to a slowing of preparation and execution of both hand and eye movements in this OA patient. This decline in performance was most apparent during the visuomotor rotations and hand movement timing was exacerbated when orienting towards off-axis, diagonal targets (i.e. where a cognitive rule is not as relevant and online sensorimotor recalibration is required). 

### Eye-hand coupling

We analyzed the impact that eye movement planning (ERT) had on hand movement planning (HRT) in both direct and decoupled situation. As such, to assess eye-hand coupling across conditions and targets for IG and the control group, we ran correlation analyses. 

IG displayed the strongest eye-hand coupling (i.e. positive correlation) for C90° towards the diagonal targets (*r* = 0.79, *p*<0.0001). Eye-hand coupling was also strong during her performance of C90° towards the horizontal targets (*r* = 0.71, *p*<0.001). Moderate positive correlations were observed for direct reaching towards both horizontal (*r* = 0.60, *p*<0.01) and diagonal targets (*r* = 0.51, *p*<0.05). ERT and HRT were not correlated for either H, S, or C180° (*p*>0.05). 

The control group displayed the strongest eye-hand coupling during direct reaching towards the diagonal targets (*r* = 0.57, *p*<0.0001). Similarly, moderate eye-hand coupling was observed during S only towards the diagonal targets (*r* = 0.47, *p*<0.0001). Moderate positive correlations were also observed for C90° towards both horizontal (*r* = 0.45) and diagonal (*r* = 0.45) targets (*p*<0.0001). Low positive correlations were observed for C180° towards horizontal (*r* = 0.24) and diagonal (*r* = 0.20) targets (*p*<0.05). No significant correlation was observed in the control group for H (*p*>0.05).

### Hand movement endpoints and trajectories

**Figure 4:**
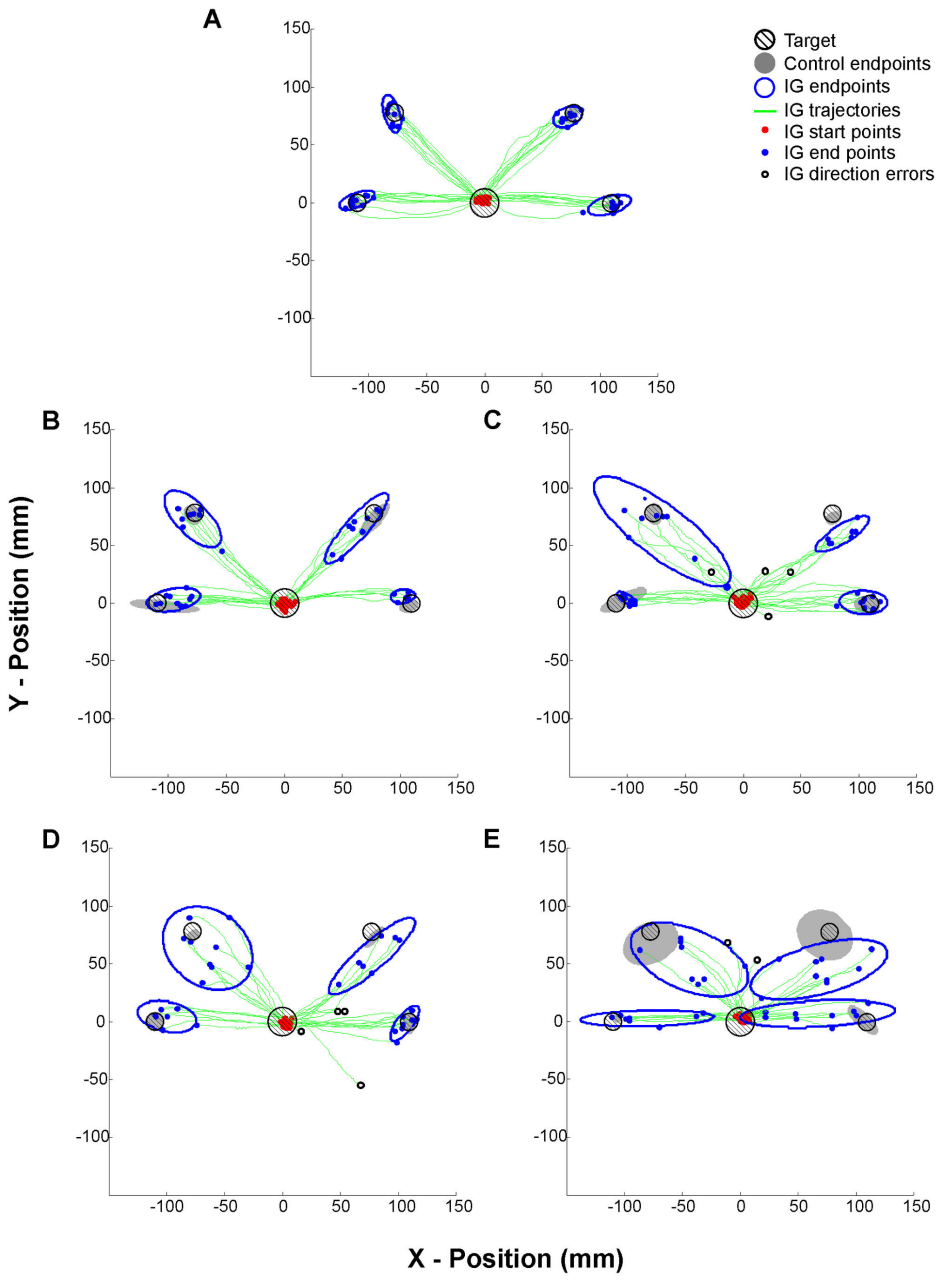
Individual hand endpoint ellipses for IG and a typical control subject. Hand movement trajectories endpoints of ballistic motor plan to four peripherally-cued targets from the home target in (A) coronal (B) horizontal (C) para-sagittal (D) coronal 180° and (E) coronal 90°. Both eye and gaze conditions were pooled for all subjects. Open and filled ellipses represent 95% confidence intervals for IG and a typical control, respectively. Trajectories (green lines), start points (closed red circles), endpoints for successful trial (closed blue circles) and direction error trials (open black circles) represent IG’s data only. Circles with cross-hatching represent starting and ending target location. Note the systematic endpoint errors for IG during decoupled reaching especially when orienting towards the diagonal (off-axis) targets, and the accurate reaching for the control subject.

**Figure 5:**
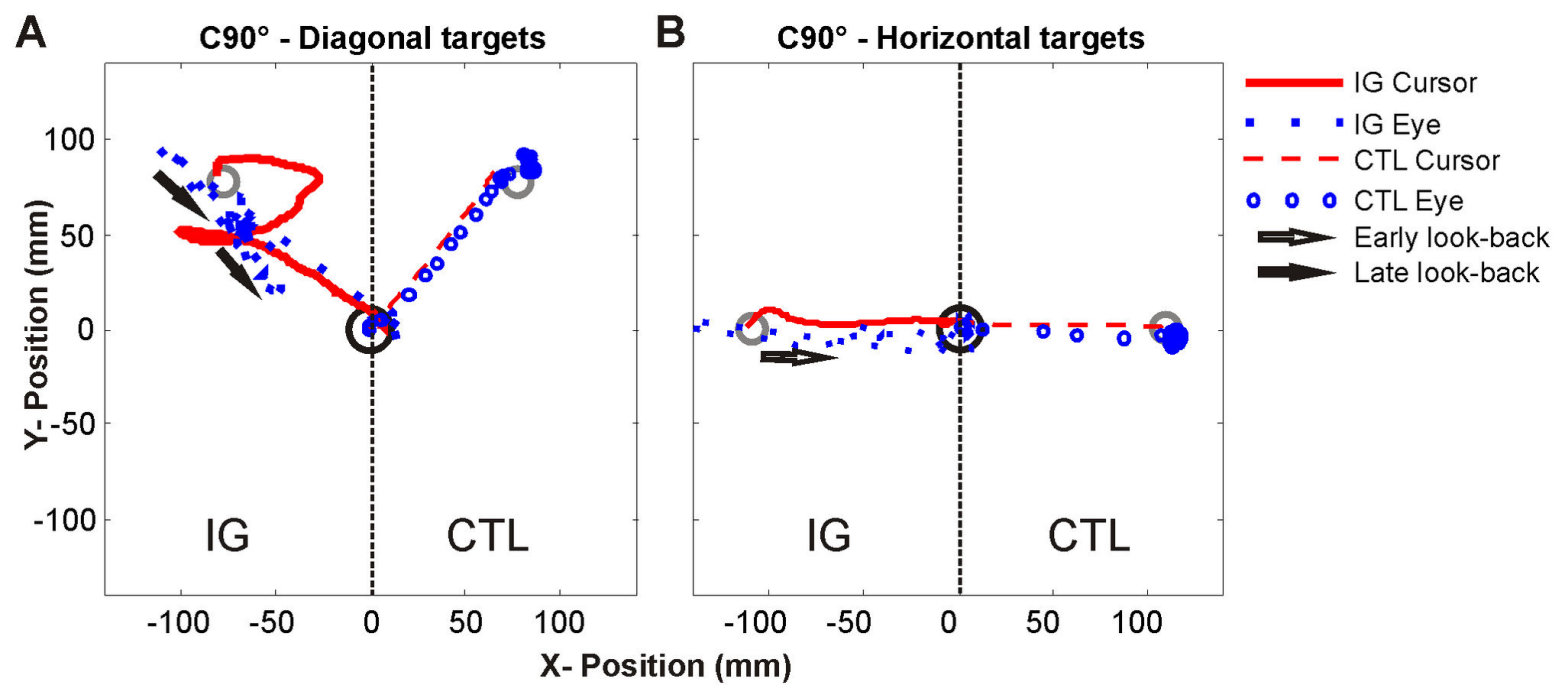
Example trials during coronal 90° condition towards diagonal and horizontal targets for IG and a typical control subject. Example hand (represented by the rotated cursor) and eye movement trajectories for the entire trial towards (A) diagonal and (B) horizontal targets. Note for IG (left side) the increase in path length towards diagonal target and the increase in late (online updating) look-backs (closed arrows), and relatively accurate hand movement trajectories accompanied by an early (priming) look-back (closed arrow) towards the horizontal target.

Analyses of hand movement endpoints of the initial ballistic motor plan, as well analyses of the entire trajectory were performed to assess the integrity of the predictive motor plan and the online correction in OA. Figure 4 displays the ballistic endpoint across all conditions for IG and for a typical control subject. For examples of full hand and eye movement trajectories between diagonal and horizontal targets during decoupled eye-hand coordination, see Figure 5.

#### Intra-subject OA details

IG displayed impaired hand endpoints and trajectories during C90°, especially towards the diagonal targets. IG performed hypometric on-axis CE (condition main effect; ANOVA, *F*_3,152_ = 8.5, *p*<0.0001) for C90° relative to H and C180° (*p*<0.001), and S (*p*<0.0001). Her off-axis CE (condition × target interaction; ANOVA, *F*_3,152_ = 3.0, *p*<0.05) was the most inaccurate in C90° relative to H specifically towards the diagonal targets (*p*<0.0001). Similarly, IG displayed the most variability in her hand endpoints (VE; condition main effect; ANOVA, *F*_3,152_ = 8.2, *p*<0.0001) for C90° relative to S and C180° (*p*<0.01), and H (*p*<0.0001). In addition, IG performed the longest trajectory (i.e. path length) depending on both condition (ANOVA, *F*_3,152_ = 4.7, *p*<0.01) and target-type (ANOVA, *F*_1,152_ = 15.3, *p*<0.0001), whereby her path length increased during C90° relative to H (*p*<0.01) and for diagonal relative to horizontal targets (*p*<0.0001). Her angle at peak velocity was the greatest (condition × target interaction; ANOVA, *F*_3,152_ = 3.5, *p*<0.001) for C90° relative to H (*p*<0.001) and C180° (*p*<0.05). When she made an initial hand direction error (DE), she took the longest to recover from the error (TTR; condition main effect; ANOVA, *F*_2,5_ = 12.9, *p*<0.05) for C90° relative to S (*p*<0.05). No comparison could be made with H as she did not perform any DE during this condition.

#### Control group

Within-group analyses were conducted on the control group for the hand position following the initial ballistic movement as well as the full hand movement trajectory. Although their initial motor commands were predominantly accurate (most landed within the target), the control group displayed differences in on-axis CE between decoupled visuomotor tasks across both target types (ANOVA, *F*_2,7_ = 7.6, *p*<0.05), and differences in off-axis CE towards horizontal targets (ANOVA, *F*_1,5_ = 8.6, p<0.05). Post-hoc comparisons revealed hypometric on-axis CE for C90° relative to C180° and greater off-axis CE for C90° compared with H (*p*<0.05). We also observed changes in hand movement path length (ANOVA, *F*_2,6_ = 10.1, *p*<0.05) between decoupled reaching tasks. However, corrected post-hoc comparisons did not reveal specific differences between conditions. No differences in hand movement precision (VE) or in the number of direction errors (DE) were observed between the conditions (*p*>0.05). 

#### OA patient versus control group

IG displayed greater hand endpoint errors relative to the control group following the ballistic portion of the movement in decoupled eye-hand situations, especially when attempting to move the cursor towards the diagonal targets (Figures 4B-E). For complete details on IG’s deficits in accuracy depending on target type, see Table 1. 

IG displayed the greatest overall increase in absolute hand endpoint errors (AE) across targets, relative to controls during C90° (*t* = 6.9, *p*′<0.01, effect size = 7.5). Specifically, IG’s ballistic motor plan was the most hypometric (gaze-biased, on-axis CE) and inaccurate (greater off-axis CE) relative to controls during C180° towards the diagonal targets (see Table 1). Similar to that observed for endpoint accuracy, IG displayed compromised hand movement trajectories during decoupled eye-hand coordination towards diagonal targets. Across target types, IG’s path length was the most compromised during C180°, relative to the control group (*t* = 12.1, *p*′<0.01, effect size = 13.1), predominantly towards the diagonal targets (Table 1). Her angle at peak velocity during C90° was the greatest across targets, relative to the control group (*t* = 6.7, *p*′<0.01, effect size = 7.3), again when attempting to accurately implement the appropriate cognitive rule towards the diagonal targets (Table 1). IG only made more initial errors in direction (DE) compared to the control group during visuomotor rotations (C180°/C90°) towards diagonal targets. Her overall time to recover from these initial direction errors (TTR), was greater than the controls (*t* = 4.0, *p*′<0.01, effect size = 4.4), especially when such errors were performed during C90° towards diagonal targets (*t* = 18.1, *p*′<0.0001, effect size = 19.6). 

In summary, differences in hand endpoint and trajectory parameters between the OA patient and the control group were observed predominantly towards diagonal targets, a situation where a strategic rule was not as useful to guide the initial motor plan of decoupled-eye hand movements.

### Eye movement errors

**Figure 6:**
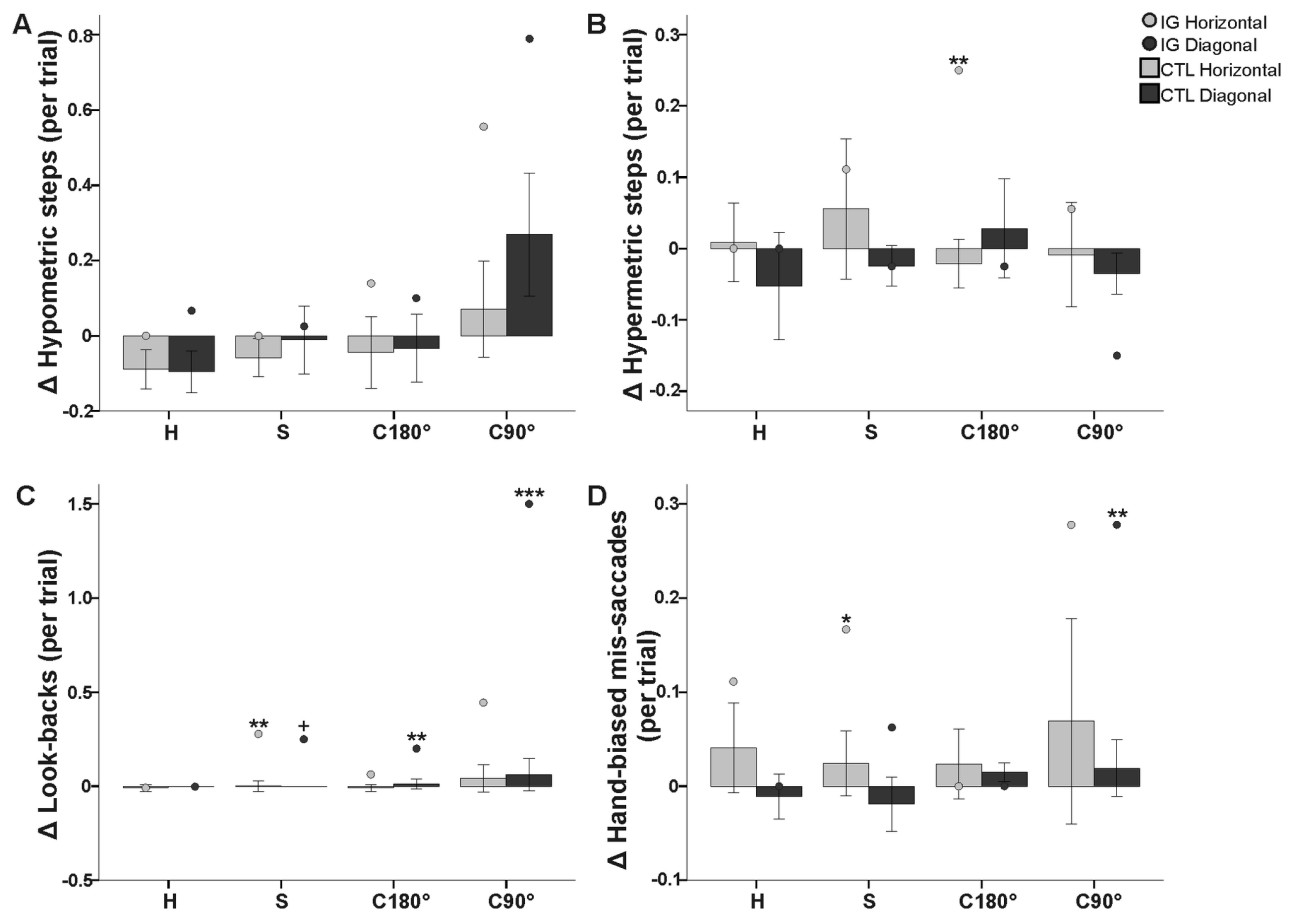
Eye errors performed by IG and the control group. Change in eye (A) hypometric steps (B) hypermetric steps (C) look-backs and (D) hand-biased mis-saccades for IG (circles) and controls (bars) for the four decoupled conditions (H = horizontal; S = para-sagittal; C180° = coronal 180°; C90° = coronal 90°) for each target type (horizontal = light gray, diagonal = dark gray) relative to standard reaching. Head-fixed and head-free conditions were pooled for all subjects. Error bars denote 95% Confidence Intervals. Note a marked increase in look-backs for IG during the performance of C90° towards diagonal targets. *p′<0.05; **p′<0.01; ***p′<0.001, corrected for multiple comparisons (Holm-Bonferroni).

Although the hand data for this OA patient demonstrated impaired performance, IG did complete all trials within the given time limit (10 seconds). The reason for her successful completion of each trial becomes clear when looking at the eye movement data. Although all subjects were instructed to foveate the peripherally-cued visual target, eye movements were not restricted. Similar to previous results observed in unilateral OA patients [11], several additional oculomotor errors were observed in this bilateral OA patient (see Figure 6). 

#### Intra-subject OA details

IG performed four basic eye movement errors in order to successfully complete the decoupled conditions. IG relied predominantly on look-backs towards the cursor during the "online updating" phase after hand movement onset (condition × target × timing interaction; ANOVA, *F*_3,262_ = 6.8, *p*<0.0001) during C90° relative to the other decoupled conditions towards the diagonal targets (*p*<0.0001). IG performed the greatest amount of hypometric steps (condition main effect; ANOVA, *F*_3,131_ = 14.7, *p*<0.0001) during C90° relative to the other decoupled conditions (*p*<0.0001). Target location influenced the number of hypermetric steps towards the computer monitor border (target main effect; ANOVA, *F*_1,131_ = 12.3, *p*<0.001), whereby she performed the greatest number of saccades towards the horizontal relative to the diagonal targets (*p*<0.001), a situation in which the computer monitor border could be useful as an accurate allocentric cue in order to complete the decoupled reach. Finally, IG also performed additional HBMS (condition main effect; ANOVA, *F*_3,131_ = 4.6, *p*<0.01) during C90° relative to C180° (*p*<0.01).

#### Control group

For the most part, the control group followed the given instructions and spontaneously performed accurate saccades, keeping their eyes on the peripherally presented visual target (Figure 6). The control group did, however, perform additional pauses (i.e. hypometric steps) during their saccades (condition main effect; ANOVA, *F*_1,6_ = 10.4, *p*<0.05) in C90° relative to C180° (*p*<0.05).

#### OA patient versus control group

IG performed more oculomotor errors than the control participants did, predominantly for C90° towards the diagonal targets (Figure 6; for specific target type details, see Table 2). 

Relative to the controls, IG primarily relied on “look-backs” towards the cursor during decoupled eye-hand coordination. Although IG performed more look-backs overall than the controls did for movements made in a para-sagittal plane (*t* = 14.0, *p*′<0.001, effect size = 15.1) and for C180° (*t* = 6.3, *p*′<0.01, effect size = 6.8), she relied predominantly on such additional saccades for C90° (*t* = 8.2, *p*′<0.001, effect size = 8.3), relying on average 1.5/trial towards diagonal targets (see Table 2). We also found that IG performed a greater number of eye movements beyond the target towards the computer monitor border than the control group did, but only during C180° (*t* = 4.0, *p*′<0.05, effect size = 4.3) and towards the horizontal targets (Table 2). Further, IG produced a greater amount of hand-biased mis-saccades (HBMS) than the controls did, specifically towards the diagonal targets (Table 2). 

In summary, during decoupled eye-hand movements, in order to successfully complete the decoupled tasks, IG made more eye movement errors than the control subjects did, particularly looking back towards the cursor (i.e. look-backs) during C90° when orienting the cursor towards the diagonal targets (see Figure 6). 

### Head movement

In order to observe behavior in a more “natural” environment, we repeated all conditions “head-free”. During such head-free conditions, we recorded the change in cumulative head movement (translation and roll, see Methods) for decoupled reaching relative to standard reaching. IG performed larger head movements (condition main effect; ANOVA, *F*_3,72_ = 41.7, *p*<0.0001) during C90° relative to the other decoupled conditions (*p*<0.0001). No differences were observed between conditions or target-type for the control group.

Although allowing free head movement did not improve hand or eye performance in either group, we did observe inter-group differences in the amount of head movement between IG and the controls during decoupled reaching. IG utilized more head movement than the controls did during the performance of C90°, for both horizontal (*t* = 14.9, *p*′<0.001, effect size = 16.1) and for diagonal (*t* = 7.2, *p*′<0.01, effect size = 7.7) targets.

In summary, although free head movements did not improve hand or eye movement performance in any participant, IG performed larger head movements in one of her more challenging conditions (C90°). 

## Discussion

The alterations in eye-hand movement performance observed in this bilateral OA patient expand our understanding of the role that the caudal SPL plays in decoupled visually-guided reaching towards objects in space [11]. Eye-hand decoupling involves the integration of a strategic motor plan with the ongoing monitoring of the current state estimate of the relative limb, gaze, and goal positions. The current study was designed to tease apart the involvement of the caudal SPL in the guidance of eye and limb during decoupled visuomotor control by varying target location as a way to require different contributions of rule integration versus ongoing movement monitoring. 

As predicted, IG demonstrated an inability to accurately update her limb position in less-categorized non-canonical situations in which she could not rely on strategic control or reliable allocentric cues. Although IG’s bilateral caudal SPL damage manifested itself as an overall impairment in online processing during decoupled eye-hand coordination, her impairment worsened in situations requiring more difficult difference vector computations (towards diagonal targets). Orienting a cursor towards diagonal targets during decoupled eye-hand coordination required a computation involving both x and y coordinates, while orienting towards horizontal targets required a computation of either x or y coordinates. In contrast to the horizontal targets, which required a trajectory that was perpendicularly aligned to the computer monitor border, the diagonal targets were not oriented directly towards a useful allocentric cue such as the corner of computer monitor border. Thus, any miscalculation of the cognitive rule required for each decoupled condition would result in an increased reliance on online updating via sensorimotor recalibration. In such a situation, IG attempted to compensate for her slow and inaccurate hand movements by utilizing several additional eye movements (e.g. looking back to the representation of current hand location from the visual target). These additional eye movements and instances of eye-hand re-coupling potentially served as a means to update the inaccurate cursor position (online) relative to the target (see example in Figure 5A). We suggest that this online updating of a decoupled difference vector [54] would be useful for the generation of corrective sub-movements [55] required to complete the decoupled movement.

### Strategic control versus sensorimotor recalibration of a decoupled limb in optic ataxia

Strategic control plays an integral role in cognitive-motor integration. However, cognitive rules alone are not sufficient; the incorporation of a rule into a motor plan must be complimented by online sensorimotor recalibration of a decoupled limb in space. Similar to previous reports in OA [11,51], IG displayed intact strategic control in the current study, particularly for well-categorized canonical movements. Meanwhile, her deficits were the greatest towards the non-canonical diagonal targets. These decrements were observed in the form of increased planning and execution time for the eye and the hand, hypometric reaching, and increased hand path length. Her deficits were markedly smaller for mappings which relied predominantly on explicit rule integration, suggesting an independent pathway for processing strategic control in non-standard visually-guided reaching (see below). 

We observed an increase in performance difficulty for patient IG relative to the control group (i.e. ES; see Figure 2) for the visuomotor rotations relative to the spatial plane dissociations and for the diagonal relative to the horizontal targets. The overall deficit across participants for C90° in particular relative to the other conditions confirms that even an adequate comprehension of a rule is not sufficient to adapt to non-canonical off-axis situations [39-41]. Specifically, the inversion of a single axis required for the computation of C90° required increases in RT as well as eye and hand movement errors across participants. However, equal performance across targets by the control group implies a specific deficit observed for IG towards the diagonal targets. In contrast, cognitive rules were previously useful for patient IG (horizontal spatial plane dissociation) after a brief training [34]. In the current study, IG’s relatively successful performance in the horizontal plane towards horizontal targets indicates that learning of a rule is sufficient in situations with reliable allocentric cues and previous experience. Therefore, a flexible balance appears to exist when learning the rules needed for strategic control versus the gradual adaptation needed for sensorimotor recalibration during decoupled eye-hand coordination. In the current situation, although all participants had an adequate comprehension of the required rule for each task, and were performing at a plateau during the task, they were not necessarily fully adapted to the different transformational manipulations. In fact, each participant had very little difficulty switching between the randomly assigned conditions (i.e. showed no after-effects), indicating that all conditions involved a strong explicit component. However, the implicit component of each condition and target type becomes apparent when observing the deficits seen as a result of OA. 

These findings suggest that an intact caudal SPL is not crucial for decoupled eye-hand movements when relying on a cognitive rule or a stereotyped motor plan (formulated via previous experience), but is integral for the realignment of decoupled vision and proprioception during novel situations or where there is no reliable rule. This latter situation likely requires the close monitoring of visual and proprioceptive information processed in this region of the brain, while the former situation could rely on intact fronto-temporal circuits for movement planning and guidance (see below).

### Potential neurological substrates for decoupled visuomotor control

Although the dorsal stream of the proposed perception-action model [56] has been well accepted as a primary network for the control of “vision for action”, it has become clear that it is not entirely functionally segregated from the control of “vision for perception” [34,35,57,58]. Instead, the dorsal stream appears to contribute to the integration of cognitive visuo-perceptual skills with complex visuomotor skills [59]. As such, it has become apparent that overlapping yet distinct cortical networks exist which control the specific components involved in decoupled eye-hand coordination. 

Notably, damage to a crucial node involved in the peripheral guidance of limb in space (caudal SPL) [11,29,31] appears to result in an inability to successfully integrate the two proposed streams. Such egocentric guidance of conflicting visual and proprioceptive information in peripheral space [11,33] and online updating required following target displacement [25-27,29,30,60] are primarily affected from dorsal stream damage. This suggests that the OA deficit includes an impaired integration of conscious awareness of eye-centered metrics with transient online representations of limb-centered metrics. Supporting the contribution of this brain region to non-standard visuomotor mapping, we have observed reduced firing rates in caudal SPL neurons in (intact) non-human primates performing similar eye-hand decoupled reaching tasks [61]. Taken together, we propose that the caudal SPL contributes to the required inhibition of the natural tendency to reach towards where one looks by monitoring the relationships between the behavioral goal and the location of the involved effectors in space, and communicating this information to frontal lobe structures involved in planning the biomechanical details of the specific movement.

The current data provide evidence of a functional spectrum from strategic control to sensorimotor recalibration of decoupled visuomotor control. Reaching in a well-learned canonical situation such as in the horizontal plane (H: when using a computer mouse), does not rely fully on an intact caudal SPL. Rather, the premotor cortex may receive indirect inputs from more *ventral* connections into the prefrontal cortex via the infero-temporal cortex or via the IPL [35], which may carry the crucial information to guide the movement. Evidence in support of these alternate connections comes from reach studies on OA patients employing a long delay between the cue and the movement [62-64]. Such connections require more processing time [65] and carry rule-based and allocentric information, which is impaired in patients such as DF with lateral occipital [66] ventral stream damage [67]. Fast, implicit guidance of a limb in peripheral space, on the other hand, relies on the combination of peripheral perception of motion with an appropriate reach vector command. It has since been suggested by [68] that the motion sensitive area MT, an area previously thought to be explicitly within the dorsal stream, and its connections with MST and IPL [69,70], may serve as an integral node in the suggested interaction between dorsal and ventral streams [35,58,71,72].

### Difference vector computation in optic ataxia

Decoupled eye-hand coordination requires ongoing overt foveal monitoring of the visual target with covert peripheral visual feedback of the limb and cursor position, along with proprioceptive feedback of the decoupled limb position. If the generated motor plan has been miscalculated, a difference vector must be continually updated online to compensate accordingly. Such a miscalculation will, in the OA patient, result in hypometric reaching (towards the direction of gaze) when reaching towards extra-foveal targets [31,73,74], proprioceptive targets [33], and foveated visual targets decoupled from the moving limb [11].

Distinct functional regions have been proposed within the SPL, with segregated areas for reaching (parietal reach region; PRR) [75,76], located within the medial bank of the IPS [77], and saccades (parietal eye fields; PEF) [78], located on the lateral bank of the IPS [77,79]. According to this segregated view, depending on the location of the lesion site, the decoupled eye-hand coordination deficits seen in OA patients could result from either impaired eye-centered coding within the PEF or a breakdown of limb-centered coding within PRR. Others have suggested the impairment in OA to result from the inability to simultaneously represent spatial orientations of decoupled end effectors when guiding a limb in peripheral visual space [80]. Thus, during decoupled eye-hand coordination OA patients may have difficulty transforming eye-centered information about the visual goal into a limb-centered motor goal [26,28,80,81]. Alternatively, evidence for reaching deficits resulting from temporary deactivation of parietal area PEc in non-human primates [47], has been thought to result from a breakdown in the *combination* of the preferred direction of eye and hand position relative to a visual target goal into a common state within the dynamic, context-dependent global tuning field of individual parietal neurons [82,83]. Thus, without a functioning SPL, the frontal cortex may not be provided with updated accurate eye-hand position signals (see 82). This breakdown of the global tuning field could explain the altered neural outputs (spikes) [61] from SPL and inputs (local field potentials) [84] into PMdr when required to formulate and maintain an accurate difference vector during decoupled eye hand coordination.

In the present study, we observed that IG had the greatest deficits for those decoupled conditions in which she could not rely on reliable allocentric aids (such as the computer monitor border) and/or simple cognitive rules (such as hand up = cursor right). When forced to rely on online updates from conflicting proprioceptive and visual information, IG displayed impaired hand movement correction when attempting to adjust for inaccurate ballistic movements. In an attempt to compensate for impaired online updating in peripheral vision, IG overtly foveated the cursor position via additional eye movements (look-backs). Such overt foveal updating of limb or target position has been previously shown to be beneficial for predicting upcoming hand movements [26,85-87]. Additional evidence for IG’s reliance on her vision for her hand movements comes from the high correlation between her eye and hand movement planning timing. During C90°, particularly towards the diagonal targets, IG’s hand movements varied relative to her eye movements to allow for efficient eye-hand coupling.

We propose that the deficits seen in this OA patient arise as a result of a failed transformation between guiding sensory information and required limb movement when strategic control is not possible. Our findings support the involvement of caudal SPL in the monitoring of gaze, limb, and target location needed for difference vector computation in decoupled reaching, a computation required for successful visuomotor transformations. The question remains if IG’s extensive previous experience has enabled neuroplasticity for decoupled visuomotor control and therefore can be accurately generalized to other OA patients. In fact, compensatory activity has been observed for IG in both occipito-temporal and in occipito-parietal regions surrounding her lesions during both immediate and delayed extra-foveal reaching [88]. However, similar observations of a reliance on strategic control during decoupled visually-guided reaching in a newly-tested OA patient MFL [11] provide evidence that IG’s deficits result from her caudal SPL lesions. Further, IG’s deficits are in line with the documented role of the caudal SPL in coding the required difference vector during decoupled reaching [61,89,90].

## Conclusions

The results of this study demonstrate that the caudal SPL is a critical component for guiding a limb to a location decoupled in space from gaze, even in situations in which one is free to foveate the visual target. These results expand our previous findings by identifying *additional* impairments in OA when orienting to off-axis diagonal targets. Such decoupled movement computations cannot be guided solely by the use of strategic control via aiming towards reliable allocentric cues. Thus we suggest that an intact caudal SPL is crucial for online updating of the decoupled limb in less-categorized non-canonical orientations in space. In addition, we suggest that a relative weighting of strategic control and sensorimotor recalibration is required depending on the type of decoupled visually-guided reach.
